# Endogenous Leucine-Rich Repeat Kinase 2 Slows Synaptic Vesicle Recycling in Striatal Neurons

**DOI:** 10.3389/fnsyn.2017.00005

**Published:** 2017-02-23

**Authors:** James W. Jr. Maas, Jing Yang, Robert H. Edwards

**Affiliations:** Departments of Neurology and Physiology, Weill Institute for Neurosciences, and Kavli Institute for Fundamental Neuroscience, UCSF School of MedicineSan Francisco, CA, USA

**Keywords:** LRRK2, endocytosis, synaptic vesicle, Parkinson’s disease, striatum

## Abstract

Dominant mutations in leucine-rich repeat kinase 2 (LRRK2) produce the most common inherited form of Parkinson’s disease (PD) but the function of LRRK2 remains poorly understood. The presynaptic role of multiple genes linked to PD including α-synuclein (α-syn) has suggested that LRRK2 may also influence neurotransmitter release, a possibility supported by recent work. However, the use of disease-associated mutants that cause toxicity complicates the analysis. To determine whether LRRK2 normally influences the synaptic vesicle, we have now used a combination of imaging and electrophysiology to study LRRK2 knockout (KO) mice. Surprisingly, we find that in hippocampal (generally excitatory) neurons, the loss of LRRK2 does not affect synaptic vesicle exocytosis, endocytosis or the mobility of α-syn. Double KO (DKO) mice lacking LRRK1 as well as LRRK2 also show no defect in transmitter release by hippocampal neurons. However, in striatal neurons, which express LRRK2 at higher levels, the loss of LRRK2 leads to modest acceleration of synaptic vesicle endocytosis. Thus, endogenous LRRK2 normally slows synaptic vesicle recycling at striatal terminals.

## Introduction

Although Parkinson’s disease (PD) is defined by the loss of dopamine neurons in the substantia nigra, accumulating evidence suggests that pathology originates at the nerve terminal (Nakata et al., 2012; Janezic et al., 2013). The protein α-synuclein (α-syn) that causes familial and idiopathic forms of PD accumulates in dystrophic axonal processes as well as somatic Lewy bodies (Polymeropoulos et al., 1997; Spillantini et al., 1997, 1998; Singleton et al., 2003). Indeed, α-syn normally localizes to the nerve terminal (George et al., 1995; Iwai et al., 1995), inhibits neurotransmitter release when over-expressed (Greten-Harrison et al., 2010; Nemani et al., 2010; Scott et al., 2010; Anwar et al., 2011; Janezic et al., 2013) and may promote formation of the SNARE complex that mediates synaptic vesicle exocytosis (Burré et al., 2010). PD-associated mutations in the endocytic proteins synaptojanin and auxilin further support a presynaptic locus for the disorder (Edvardson et al., 2012; Quadri et al., 2013).

In contrast to the rare mutations in α-syn, synaptojanin and auxilin, dominant mutations in leucine-rich repeat kinase 2 (LRRK2) produce the most common form of inherited PD and up to 10% of sporadic disease (Healy et al., 2008). LRRK2 is a multi-domain protein with roles reported in the endolysosomal pathway (Dodson et al., 2012; Gómez-Suaga et al., 2014), autophagy (Orenstein et al., 2013), translation (Martin et al., 2014) and transmitter release. Since the pathology resulting from LRRK2 mutations usually involves deposition of α-syn (Giasson et al., 2006), it is possible that LRRK2, like α-syn, modulates neurotransmitter release, and that mutant LRRK2 causes disease through a presynaptic mechanism, possibly involving synuclein.

LRRK2 localizes to the nerve terminal, but in contrast to α-syn, is not exclusively presynaptic (Biskup et al., 2006). Nonetheless, LRRK2 has been reported to interact with multiple presynaptic proteins, including NEM-sensitive factor (NSF) and the t-SNARE syntaxin-1, and may bind to purified synaptic vesicles, whereas loss of LRRK2 alters the density and size of synaptic vesicles (Piccoli et al., 2011, 2014; Cirnaru et al., 2014; Arranz et al., 2015; Belluzzi et al., 2016). LRRK2 has also been reported to influence synaptic physiology. Mice overexpressing wild type (WT) or mutant LRRK2 show opposing effects on dopamine release in the striatum (Li et al., 2009, 2010), suggesting a presynaptic site of action. Modest, transient changes in synaptic activity have been reported in mice expressing mutant LRRK2 and LRRK2 knockout (KO) mice, but these may reflect the modification of dendritic spines (Parisiadou et al., 2014; Matikainen-Ankney et al., 2016). Moreover, another study was unable to demonstrate any purely presynaptic abnormalities in LRRK2 KO mice or mice over-expressing WT LRRK2 (Beccano-Kelly et al., 2015). In cultured neurons, RNAi-mediated silencing of LRRK2 has been reported to promote synaptic vesicle exocytosis (Piccoli et al., 2011), but other work has suggested changes in endocytosis due to loss of LRRK2 or expression of mutant LRRK2 (Shin et al., 2008; Matta et al., 2012; Arranz et al., 2015; Belluzzi et al., 2016).

Since PD-associated mutants can produce toxicity that complicates the analysis of presynaptic function, we have now used KO mice and a combination of optical and electrophysiological techniques to elucidate the role of LRRK2 in the synaptic vesicle cycle. We demonstrate that loss of LRRK2 does not alter basal synaptic transmission, and does not alter the synaptic vesicle cycle in cultured glutamatergic hippocampal neurons. Further, the loss of LRRK2 is not compensated for by residual LRRK1. However, in striatal neurons, which express endogenous LRRK2 at high levels, loss of LRRK2 leads to a modest acceleration of endocytosis. Thus, LRRK2 normally inhibits synaptic vesicle recycling in striatal neurons.

## Materials and Methods

### Animals

This study was carried out in strict accordance with the recommendations in the Guide for the Care and Use of Laboratory Animals of the National Institutes of Health. The protocol was approved by the Institutional Animal Care and Use Committee of the University of California, San Francisco (protocol number: AN109510). All efforts were made to minimize suffering. LRRK1 KO mice were generated by the Michael J. Fox Foundation and both LRRK1 and 2 KO (Lin et al., 2009) mice obtained from Jackson Labs. Due to reduced survival on the C57Bl/6 background, heterozygous LRRK1 mice on this background were crossed with FVB mice to produce LRRK1 KO mice. LRRK1 KO and WT littermate controls were generated from heterozygous LRRK1 parents. We used LRRK1 heterozygous/LRRK2 KO breeding pairs to produce LRRK1 KO/LRRK2 KO mice and LRRK1 WT/LRRK2 KO littermates as control. Animals were genotyped by PCR.

### Neuronal Culture and Transfection

To produce mouse hippocampal cultures, postnatal day 0 hippocampus was digested in papain (20 U/ml, Worthington), washed, triturated and plated on glass coverslips pre-coated with poly-L-lysine at a density of 700 cells/mm^2^. Neurons were plated in minimal essential media containing 5% FBS, B27 supplement (Gibco), 21 mm glucose, 2 mM glutamax and Mito+ serum extender (VWR). Twenty-four hours after plating, 75% of the medium was removed and replaced with medium containing Neurobasal, B27 supplement, and 2 mM glutamax. After 7 days in culture, one third of the medium was exchanged, and 4 μM cytosine arabinoside added to prevent glial proliferation. Neurons were transfected using calcium phosphate at 6–7 days *in vitro* (DIV). To produce mixed cortical/striatal neuron cultures, the medial ganglionic eminence and cortex were isolated from embryonic day 16 mouse embryos, digested in trypsin (2.5%, Gibco) and DNase I (10 mg/ml, Roche), washed and triturated. Striatal neurons were transfected by electroporation (Amaxa), mixed with untransfected cortical neurons (3:2 striatal:cortical) and plated on glass coverslips pre-coated with poly-L-lysine at a density of 1100 cells/mm^2^ in Neurobasal medium containing B27 supplement and 2 mM glutamax. Medium was exchanged and cytosine arabinoside added as described for hippocampal cultures.

### Imaging

Hippocampal neurons were transfected with the pH-sensitive GFP variant superecliptic pHluorin, targeted to the lumenal domain of synaptic vesicles by insertion into the large lumenal loop of vesicular glutamate transporter 1 (VGLUT1-pHluorin; Voglmaier et al., 2006). Striatal neurons were transfected with the vesicular GABA transporter (VGAT) tagged with pHluorin at the lumenal/extracellular C-terminus (VGAT-pHluorin; Santos et al., 2013). Neurons were imaged between DIV 14–17, as previously described (Nemani et al., 2010). Briefly, coverslips containing neurons were placed in an RC-21BRFS imaging chamber containing bipolar stimulating electrodes (Warner Instruments) in closed configuration. Neurons were continuously perfused (1 ml/min) at room temperature with Tyrode’s solution (in mM: 119 NaCl, 2.5 KCl, 2 CaCl_2_, 2 MgCl_2_, 30 glucose and 25 HEPES, pH 7.4) containing glutamate receptor antagonists 6-cyano-7 nitroquinoxaline-2, 3-dione (CNQX; 10 μM) and D, L-2-amino-5-phosphonovaleric acid (APV; 50 μM) and stimulated using trains of 1 ms current pulses to yield fields of 5–10 V/cm. We usually delivered a train of 600 stimuli at 10 Hz for 60 s, but in some experiments we delivered shorter trains at alternate frequencies, as indicated in the text. NH_4_Cl buffer (in mM: 69 NaCl, 2.5 KCl, 2 MgCl_2_, 2 CaCl_2_, 50 NH_4_Cl, 30 glucose, 25 HEPES, pH 7.4) was used to reveal total pHluorin fluorescence. Images were collected under epifluorescence illumination using a Nikon TE300 inverted microscope and xenon lamp with ET470/40 nm excitation and ET525/50 nm emission bandpass filters, a 63× 1.2 N.A. water objective, and a EM-CCD camera (Photometrics QuantEM:512SC) controlled by Metamorph software.

Initial rate of fluorescence increase was calculated by determining the slope of a line through the data point immediately prior to stimulation and the first two points during stimulation. To specifically assess exocytosis, we stimulated neurons at 10 or 20 Hz for 150 s in the presence of bafilomycin (0.6 μM, diluted from 1000× stock solutions in DMSO). Initial exocytic rate in bafilomycin was calculated by determining the slope of a line through the data points in the first 10 s after start of stimulation, when the increase in fluorescence is linear.

Endocytosis rate was calculated by normalizing curves to the peak response at the end of stimulation and fitting a single exponential decay curve to determine the decay constant, *τ*.

For destaining experiments, neurons were loaded by stimulation at 10 Hz for 2 min in 15 μM FM4–64 (ThermoFisher) in Tyrode’s solution, allowed to recover for 2 min, washed briefly with Tyrode’s solution containing 1 mM Advasep-7 (Biotium) followed by a longer wash with Tyrode’s solution, and images were collected as above using ET 470/40 nm excitation and 650 longpass emission filters. Curves were normalized to the initial peak fluorescence and fitted with a single exponential decay curve to determine the time constant *τ*, reflecting the loss of dye and by inference, the rate of exocytosis.

### Fluorescence Recovery after Photobleaching

Neurons were imaged between DIV 14–17 in Tyrode’s solution with 10 μM CNQX and 50 μM APV at 37°C using a Zeiss LSM 510 confocal microscope with 63× oil immersion objective (NA 1.4) and 488 nm argon laser line. The pinhole was set to 2.5 μm to collect light from the entire bouton and images were collected every 400 ms at 1.5% transmission to reduce bleaching. For photobleaching, 40 consecutive scans were performed over 1 s at 100% transmission. The curves for fluorescence recovery after photobleaching (FRAP) were fit to a single exponential to calculate the time constant for recovery, *τ*.

### Electrophysiology

Acute hippocampal slices were prepared from 3 week old mice. Three-hundred and fifty micrometer hippocampal slices were cut with a vibratome in aerated slicing solution (in mM, 228 sucrose, 2.5 KCl, 1 NaH_2_PO_4_, 7 MgSO_4_, 0.5 CaCl_2_, 26 NaHCO_3_ and 11 dextrose) at 4°C, transferred to artificial cerebrospinal fluid (aCSF; in mM, 119 NaCl, 2.5 KCl, 1.3 MgSO_4_, 2.5 CaCl_2_, 26 NaHCO_3_, 1 NaH_2_PO_4_ and 11 dextrose adjusted to 315 mosmol/L and pH 7.4) at 35°C and allowed to recover for a minimum of 1 h. Slices were visualized with an upright infrared differential interference contrast microscope and perfused with standard aCSF at room temperature. Stimulation (100 μs) pulse was delivered with a bipolar metal electrode (MX21AEW; FHC). Synaptic strength was quantified as the initial slope of field potentials recorded with aCSF-filled microelectrodes (1–2 MΩ).

### Statistical Analysis

Data are presented as mean ± SEM. For experiments involving multiple measurements among genotypes, we utilized 2-way analysis of variance (ANOVA), followed by *post hoc* Bonferonni tests. For experiments involving a single comparison between genotypes, we used two-tailed, unpaired *t*-tests. All statistics were calculated using Graphpad Prism.

## Results

### LRRK2 and Basal Synaptic Transmission

**Figure 1:**
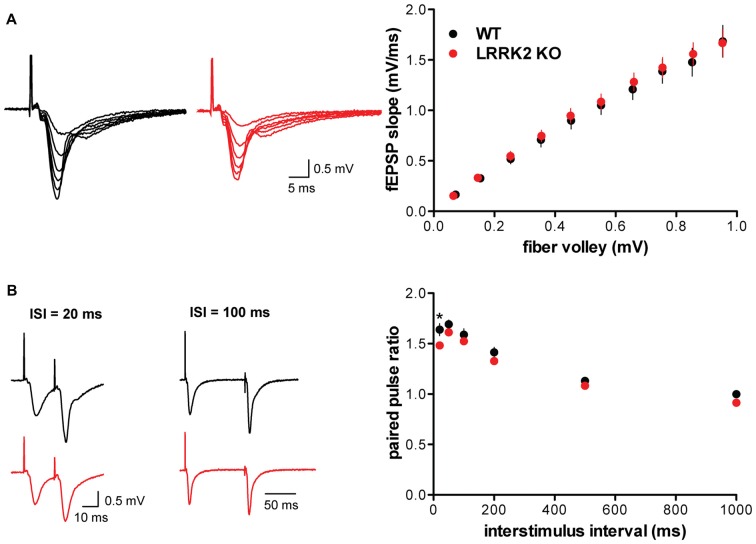
**Loss of leucine-rich repeat kinase 2 (LRRK2) does not alter basal synaptic transmission. (A)** Representative traces (left) and input-output curve (right) of fEPSP slope normalized to fiber volley in hippocampal slices from wild type (WT; black) and LRRK2 knock-out (KO; red) mice (*F*_(1,306)_ = 0.66, *p* = 0.42, *n* = 15–17 slices from three animals per genotype). **(B)** Left, representative traces of field potentials from WT (black) and LRRK2 KO (red) slices stimulated at interstimulus intervals (ISI) of 20 and 100 ms. Right, paired pulse ratio of fEPSP in WT and LRRK2 KO hippocampal slices differs only at the 20 ms ISI (*F*_(1,186)_ = 17.53, *p* < 0.0001, *p < 0.05 by post-test, *n* = 16–17 slices from three animals per genotype). The data represent mean ± SEM.

To determine whether the loss of LRRK2 affects synaptic transmission, we used field recordings in hippocampal slices. We first examined the input-output curve, normalizing the slope of the field potential to the strength of the fiber volley. Importantly, the loss of LRRK2 does not influence the evoked synaptic response (Figure 1A). We then assessed release probability using the paired-pulse ratio (PPR), which shows no effect of the LRRK2 KO except at the 20 ms interstimulus interval, suggesting little or no effect on neurotransmitter release (Figure 1B).

### Loss of LRRK2 Does Not Alter the Synaptic Vesicle Cycle

**Figure 2:**
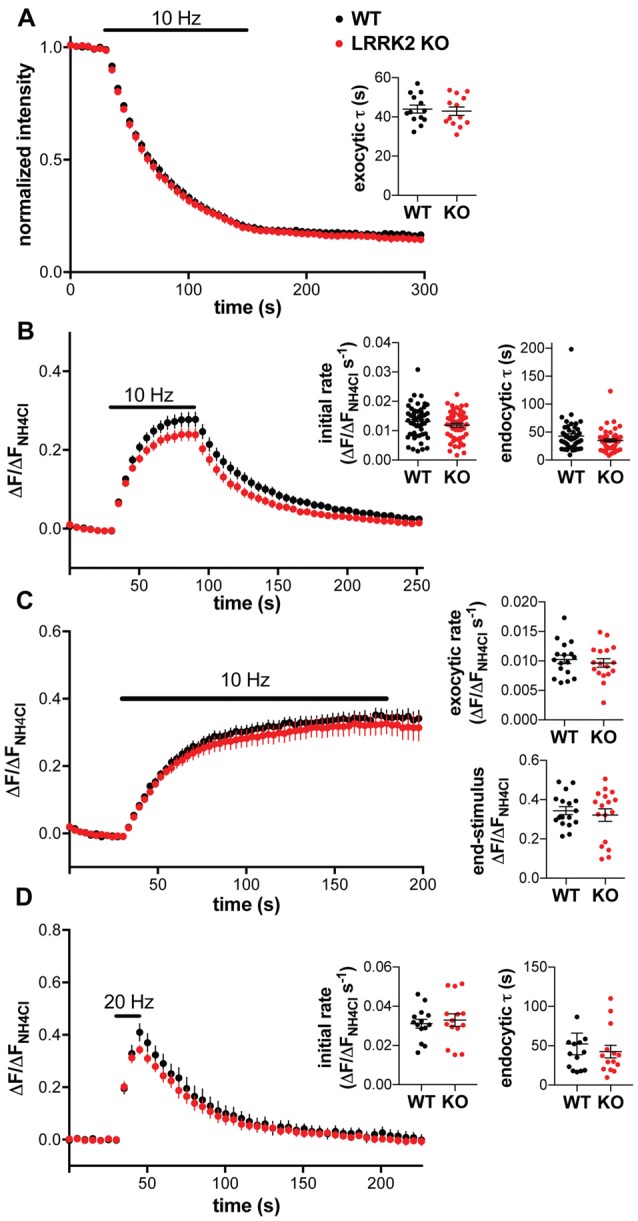
**Loss of LRRK2 does not alter synaptic vesicle exocytosis or endocytosis in hippocampal neurons. (A)** Hippocampal neurons prepared from WT and LRRK2 KO mice were loaded with FM4–64 and destained by stimulation at 10 Hz for 120 s. Inset shows the time constant for destaining (*p* = 0.72, *n* = 13 coverslips per genotype). **(B)** Response of VGLUT1-pHluorin to 10 Hz stimulation for 60 s in transfected WT or LRRK2 KO hippocampal neurons, normalized to total VGLUT1-pHluorin revealed by alkalinization with 50 mM NH_4_Cl. Insets show the initial rate of fluorescence increase (*p* = 0.24) and the time constant for endocytosis (*p* = 0.11). *n* = 50–52 coverslips per genotype. **(C)** Response of VGLUT1-pHluorin to 10 Hz stimulation for 150 s in the presence of bafilomycin in transfected WT or LRRK2 KO hippocampal neurons. Insets show the initial exocytic rate (*p* = 0.53) and the fluorescence at the end of stimulation (*p* = 0.56). *n* = 17 coverslips per genotype. **(D)** Response of VGLUT1-pHluorin to 20 Hz stimulation for 15 s in transfected WT or LRRK2 KO hippocampal neurons. Insets show the initial rate of fluorescence increase (*p* = 0.64) and the time constant for endocytosis (*p* = 0.54). *n* = 14 coverslips per genotype The data represent mean ± SEM.

To characterize the role of LRRK2 in synaptic vesicle cycling, we first used the styryl dye FM4–64. Like FM1–43, the red-shifted FM4–64 can be loaded into recycling synaptic vesicles by stimulation, and the subsequent stimulation-dependent destaining used to monitor synaptic vesicle exocytosis (Fernández-Alfonso and Ryan, 2004). Surprisingly, hippocampal neurons from WT and LRRK2 KO mice show no difference in either the rate or extent of destaining (Figure 2A), suggesting no role for LRRK2 in either synaptic vesicle exocytosis or the size of the recycling pool.

To monitor synaptic vesicle cycling with a more sensitive reporter that also has the potential to assess endocytosis, we used the pH-sensitive GFP variant superecliptic pHluorin, targeted to the lumenal domain of synaptic vesicles by insertion into a lumenal loop of vesicular glutamate transporter 1 (VGLUT1-pHluorin; Voglmaier et al., 2006). Quenched at the low pH of synaptic vesicles, the fluorescence increases with exocytosis and declines with the reacidification that follows endocytosis (Miesenböck et al., 1998). We transfected this reporter into hippocampal neurons from WT and LRRK2 KO mice, and imaged the change in fluorescence in response to stimulation, normalizing to the total pool of VGLUT1-pHluorin revealed by alkalinization in NH_4_Cl (Δ*F*_NH_4_Cl_). We again observed no difference between WT and LRRK2 KO neurons in the response to stimulation, including the initial rate of response or subsequent endocytosis (Figure 2B), with values very similar to those previously reported (Voglmaier et al., 2006; Nemani et al., 2010; Armbruster et al., 2013). However, the initial rate of fluorescence increase reflects the net contribution of both exocytosis and endocytosis. To more specifically assess the impact of LRRK2 on synaptic vesicle exocytosis, we repeated the experiments in the presence of the proton pump inhibitor bafilomycin to prevent the quenching of VGLUT1-pHluorin fluorescence by reacidification of internalized vesicles, so that the response to stimulation reflects only exocytosis (Figure 2C). In the presence of bafilomycin, there was no difference between WT and LRRK2 KO neurons in the rate or extent of exocytosis, which reflects the total pool of recycling vesicles, consistent with the lack of difference in initial rates observed without bafilomycin (Figures 2B,C).

### LRRK2 Does Not Regulate the Mobility of α-Synuclein

**Figure 3:**
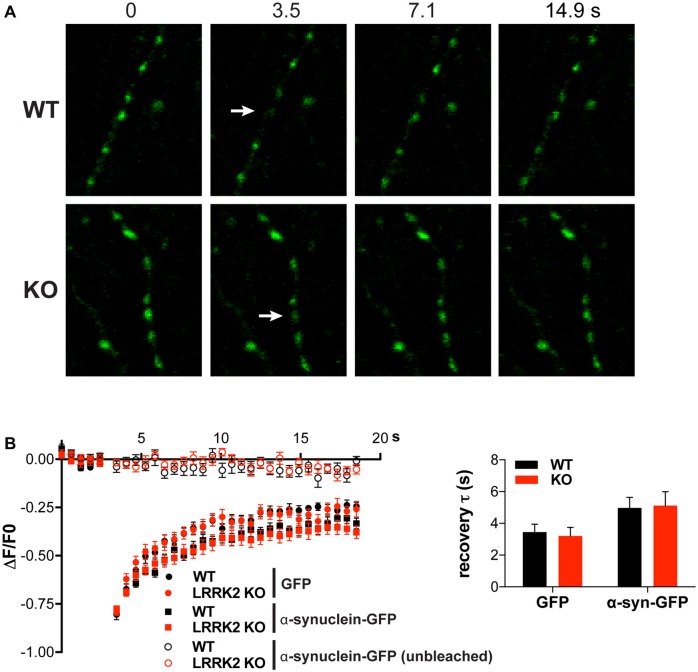
**Loss of LRRK2 does not alter the mobility of α-synuclein (α-syn). (A)** In either WT or LRRK2 KO hippocampal neurons, a single presynaptic bouton expressing α-syn-GFP was photobleached (white arrow) and the recovery of fluorescence monitored at 400 ms intervals. **(B)** Hippocampal neurons from either WT or LRRK2 KO mice were transfected with GFP or α-syn-GFP and the average change in fluorescence recorded before and after photobleaching (boutons bleached at 3.5 s after start of imaging). Nearby unbleached boutons expressing or α-syn-GFP are shown as control. Inset shows the time constant for fluorescence recovery (WT vs. KO: *F*_(1,60)_ = 0.09, *p* = 0.77; GFP vs. α-syn-GFP: *F*_(1,60)_ = 6.68, *p* = 0.012; *n* = 16 boutons per genotype). The data represent mean ± SEM.

LRRK2 may contribute to a presynaptic form of degeneration independent of synaptic vesicle cycling. Since association with membranes can influence the misfolding and aggregation of α-syn (Davidson et al., 1998; Lee et al., 2002), we used FRAP to assess a role for LRRK2 in its mobility, which presumably reflects membrane association. In previous work, we showed that after photobleaching a single presynaptic bouton, α-syn-GFP fluorescence recovers much more rapidly than the fluorescence of integral membrane proteins but more slowly than that of soluble GFP (Figures 3A,B), suggesting that α-syn binds specifically but weakly to presynaptic structures (Fortin et al., 2004; Unni et al., 2010). Consistent with this interpretation, the A30P mutation disrupts presynaptic accumulation of α-syn and accelerates recovery from photobleaching to the rate observed for soluble GFP (Fortin et al., 2004, 2005). We now find that the rate and extent of recovery for α-syn-GFP shows no difference between hippocampal neurons from WT and LRRK2 KO mice (Figures 3A,B). LRRK2 thus does not appear to influence the mobility of α-syn at the presynaptic terminal, and by inference, the association of α-syn with presynaptic membranes.

### Loss of LRRK1 Does Not Alter Synaptic Vesicle Release

**Figure 4:**
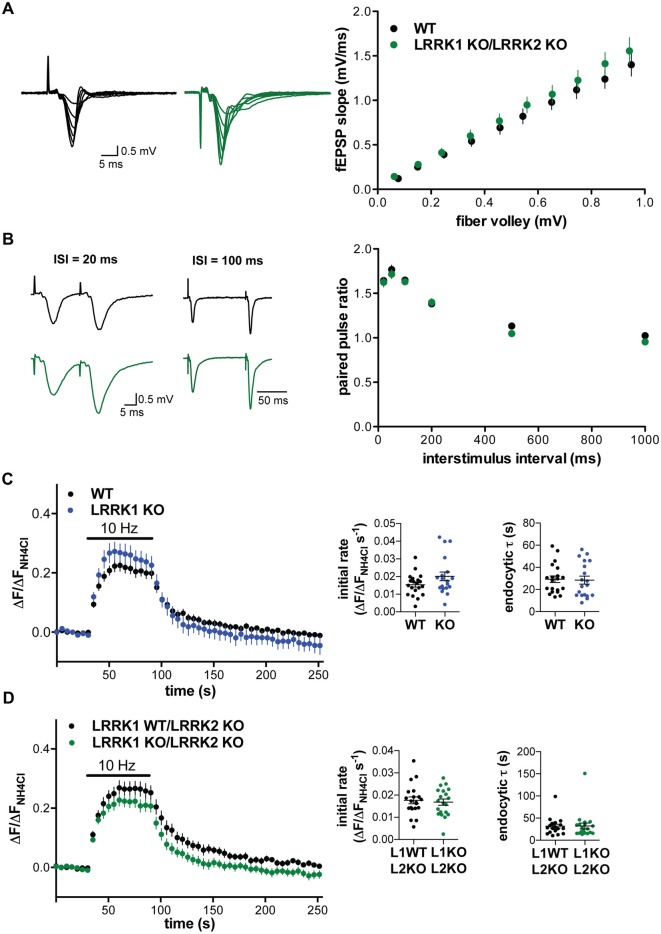
**Loss of LRRK1 and LRRK2 does not alter basal synaptic transmission or the synaptic vesicle cycle. (A)** Representative traces (left) and input-output curve (right) of fEPSP slope normalized to fiber volley in hippocampal slices from WT (black) and LRRK1 KO/LRRK2 KO (green) mice (*F*_(1,271)_ = 5.094, *p* = 0.025 *n* = 15 slices from 2 to 3 animals per genotype). **(B)** Left, representative traces of field potentials from WT (black) and LRRK1 KO/LRRK2 KO (green) slices stimulated at ISI of 20 and 100 ms. Right, paired pulse ratio does not differ between WT and LRRK1 KO/LRRK2 KO hippocampal slices (*F*_(1,168)_ = 2.99, *p* = 0.086, *n* = 15 slices from 2 to 3 animals per genotype). Fluorescence of VGLUT1-pHluorin in response to 10 Hz stimulation for 60 s in WT or LRRK1 KO **(C)** and LRRK1 WT/LRRK2 KO or LRRK1 KO/LRRK2 KO hippocampal neurons **(D)**. Insets show the initial rate of fluorescence increase (*p* = 0.09 for **C**, 0.69 for **D**) and the time constant for endocytosis (*p* = 0.86 for **C**, 0.98 for **D**; *n* = 19–20 coverslips per genotype). The data represent mean ± SEM.

Since loss of the single LRRK protein in *Drosophila* impairs synaptic vesicle endocytosis (Matta et al., 2012), we considered the possibility that the two mammalian isoforms might be redundant. We therefore generated double KO (DKO) mice lacking both isoforms, and performed field recordings in hippocampal slices. The input-output curve comparing the slope of the field potential to the strength of the fiber volley in LRRK1/2 DKO slices showed a significant main effect of genotype vs. WT (2-way ANOVA, *F*_(1,271)_ = 5.094, *p* = 0.025), but *post hoc* tests showed no significant differences at any stimulation intensity (Figure 4A). Consistent with the absence of change in baseline synaptic transmission, release probability assessed using the PPR shows no difference from WT in the LRRK1/2 DKO (Figure 4B). We also examined the LRRK1/2 DKO using cultured hippocampal neurons transfected with VGLUT1-pHluorin. Loss of LRRK1 does not by itself alter the initial rate of fluorescence increase or compensatory post-stimulus endocytosis (Figure 4C). We then examined the effect of LRRK1 inactivation in the LRRK2 KO background (LRRK1WT/LRRK2 KO vs. LRRK1 KO/LRRK2 KO). Loss of both isoforms also failed to alter either the fluorescence increase or decrease after stimulation (Figure 4D), demonstrating that LRRK1 does not compensate for the loss of LRRK2.

### Loss of LRRK2 Accelerates Endocytosis in Striatal Neurons

**Figure 5:**
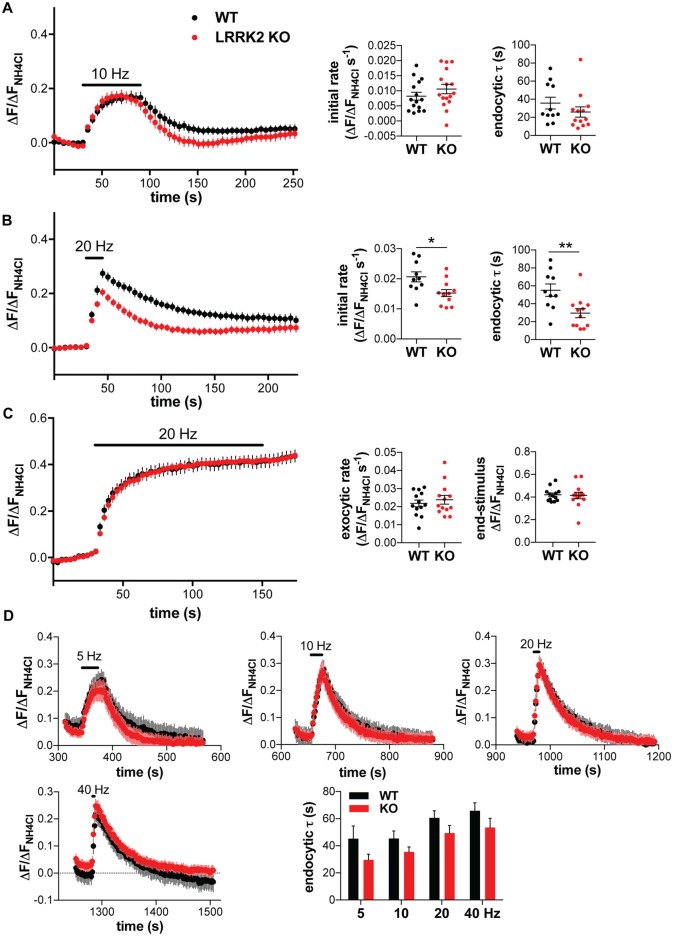
**Loss of LRRK2 accelerates synaptic vesicle endocytosis in striatal neurons.** Response of vesicular GABA transporter (VGAT)-pHluorin to stimulation at 10 Hz for 60 s **(A)** or 20 Hz for 15 s **(B)** in transfected WT or LRRK2 KO striatal neurons. Insets show initial rate of fluorescence increase (*p* = 0.25 for **A**, 0.014 for **B**) and the time constant for endocytosis (*p* = 0.26 for **A**, 0.0064 for **B**). *n* = 15–16 coverslips per genotype for **(A)**, 10–12 coverslips per genotype for **(B)**; *p < 0.05 by *t*-test, **p < 0.01 by *t*-test. **(C)** Response of VGAT-pHluorin to 20 Hz stimulation for 150 s in the presence of bafilomycin in transfected WT or LRRK2 KO striatal neurons. Insets show the initial exocytic rate (*p* = 0.52) and the fluorescence at the end of stimulation (*p* = 0.87). *n* = 13 coverslips per genotype. **(D)** Response of VGAT-pHluorin to sequential trains of 200 stimuli at increasing frequency (5 Hz for 40 s, 10 Hz for 20 s, 20 Hz for 10 s and 40 Hz for 5 s) in transfected WT or LRRK2 KO striatal neurons. The time constant for endocytosis demonstrates a significant main effect of genotype (*F*_(1,88)_ = 08.703, *p* = 0.0041) and stimulus frequency (*F*_(3,88)_ = 6.753, *p* < 0.0004; *n* = 11–13 coverslips per genotype). The data represent mean ± SEM.

Several studies have indicated high levels of native LRRK2 expression in the striatum (Melrose et al., 2006; Higashi et al., 2007; Westerlund et al., 2008; Giesert et al., 2013), which contains predominantly GABA neurons, suggesting that these cells may be more affected by the loss of LRRK2. Thus, we also examined the synaptic vesicle cycle in cultured striatal neurons from WT and LRRK2 KO mice, using the VGAT tagged with pHluorin at the lumenal/extracellular C-terminus (VGAT-pHluorin; Santos et al., 2013). The rate of both fluorescence increase and compensatory endocytosis after stimulation at 10 Hz for 60 s did not significantly change with loss of LRRK2, but endocytosis showed a trend to acceleration (Figure 5A). Previous studies using a shorter train of higher frequency stimulation (20 Hz for 15 s) have suggested that loss of LRRK2 inhibits endocytosis in rat striatal neurons (Arranz et al., 2015). Surprisingly, stimulation of mouse striatal neurons transfected with VGAT-pHluorin at 20 Hz for 15 s shows a significantly slower rate of fluorescence increase in LRRK2 KO than WT neurons, with a clear acceleration of compensatory endocytosis after the stimulus (Figure 5B). To determine whether loss of LRRK2 might also affect exocytosis in striatal neurons, we stimulated at 20 Hz in the presence of bafilomycin. Under these conditions, there was no difference in the rate or extent of exocytosis between WT and LRRK2 KO neurons (Figure 5C), supporting our conclusion that loss of LRRK2 does not directly affect the rate of exocytosis. Since stimulation at 20 Hz seemed to reveal a bigger difference in endocytosis between genotypes, we stimulated with sequential trains of 200 stimuli at 5–40 Hz (Figure 5D). As previously reported for hippocampal neurons, endocytosis was slower at higher stimulus frequencies in both genotypes (*F*_(3,88)_ = 6.753, *p* < 0.0004; Armbruster et al., 2013; Kononenko et al., 2014; Figure 5D). However, LRRK2 genotype affected the rate of endocytosis across all stimulation frequencies (*F*_(1,88)_ = 08.703, *p* = 0.0041). Although fluorescence appears to decay below baseline after 5 Hz stimulation, the baseline is in fact elevated due to incomplete decay after an initial stimulus (data not shown): in striatal neurons, fluorescence often fails to recover fully to the pre-stimulus baseline after a single stimulus train (Figures 5A,B); with subsequent stimulation, the fluorescence decays to the original pre-stimulus baseline (Figure 5D). In contrast to striatal neurons, stimulation of hippocampal neurons transfected with VGLUT1-pHluorin at 20 Hz for 15 s showed no difference between genotype in either the rate of fluorescence increase or post-stimulus endocytosis (Figure 2D). Thus, the effect of LRRK2 on rate of endocytosis depends on neuron identity, not stimulation frequency.

## Discussion

The results indicate that LRRK2 has a modest inhibitory effect on endocytosis in striatal but not hippocampal neurons. In hippocampal slices, loss of LRRK2 has no consistent effect on basal synaptic transmission. We also failed to detect any change in synaptic vesicle exo- or endocytosis in hippocampal neurons from LRRK2 KO mice using both FM dye and more sensitive pHluorin-based reporters. The analysis of LRRK1/2 DKO mice shows that residual LRRK1 does not compensate for the loss of LRRK2. Further, LRRK2 does not affect the mobility of α-syn and, by inference, its association with presynaptic membranes (Fortin et al., 2004; Unni et al., 2010). In striatal neurons, however, loss of LRRK2 leads to acceleration of endocytosis.

Why would we observe a bigger role for LRRK2 in striatal than hippocampal neurons? Striatal spiny projection neurons express LRRK2 at particularly high levels (Melrose et al., 2006; Higashi et al., 2007; Westerlund et al., 2008; Giesert et al., 2013). An effect on endocytosis is thus more likely to become apparent in striatal than hippocampal neurons, which express LRRK2 at lower levels. We cannot exclude the possibility that the difference reflects the use of different reporters (VGLUT1- vs. VGAT-pHluorin). However, previous work has shown that these two reporters have identical kinetics in hippocampal neurons (Santos et al., 2013). Thus, the differences we observe are unlikely to reflect the use of different reporters. Furthermore, experiments to assess the kinetics of VGAT-pHluorin specifically in GABAergic neurons of the hippocampus are technically challenging since they represent only 6% of the neurons in hippocampal cultures (Benson et al., 1994). The results thus support a larger role for LRRK2 in striatal neurons, which are largely GABAergic, than in hippocampal neurons, which are largely glutamatergic, presumably due to differential expression of LRRK2.

Studies to date on the role of LRRK2 in modulating presynaptic function have reported contradictory results. In the first studies suggesting a synaptic role for LRRK2, transgenic overexpression of WT LRRK2 enhanced striatal dopamine release, whereas overexpression of mutant LRRK2 had the opposite effect (Li et al., 2009, 2010). In the LRRK2 KO, evoked dopamine release was unaffected (Hinkle et al., 2012). However, changes in the amount of dopamine released could reflect alterations that do not involve the release machinery. Electrophysiologic recording from hippocampal slices of aged transgenic mice overexpressing G2019S LRRK2 has shown enhanced basal transmission, but without effect on paired pulse facilitation, suggesting a post- rather than presynaptic change (Sweet et al., 2015). Our study now provides the first analysis of the LRRK2 KO in hippocampal slices, with no clear effects on baseline excitatory transmission. In accord with these results, striatal slices from LRRK2 KO mice have shown no change in spontaneous release or paired pulse ratio (Parisiadou et al., 2014; Beccano-Kelly et al., 2015).

To image release directly, we and others have used cultured neurons. Using pHluorin-based reporters, overexpression of WT or mutant LRRK2 (G2019S) has been reported to slow endocytosis in cultured rat hippocampal neurons, without affecting exocytosis (Shin et al., 2008). In contrast, cortical neurons from transgenic mice overexpressing mutant LRRK2 (G2019S), have shown modest acceleration of endocytosis (Belluzzi et al., 2016). Although it is difficult to reconcile these results, over-expression of mutant LRRK2 also exerts toxicity (MacLeod et al., 2006; Smith et al., 2006). To study the function of LRRK2 without this complication, several groups have knocked down LRRK2 using siRNA, finding both increased paired-pulse depression (Piccoli et al., 2011) and reduced endocytosis without effects on exocytosis (Shin et al., 2008). Off-target effects of the RNAi may contribute to the apparent discrepancies. Consistent with the majority of these results, we find that LRRK2 does not modulate synaptic vesicle exocytosis. We cannot exclude specific effects on the readily releasable pool, but analysis of the recycling pool shows no changes that would suggest such a specific effect.

Avoiding the problems associated with overexpression and RNAi, analysis of *Drosophila* lacking the single LRRK gene has shown defects in endocytosis. LRRK apparently phosphorylates the BAR (bin-amphiphysin-rev) domain protein endophilin, which has a well established role in endocytosis (Matta et al., 2012). Consistent with the work in *Drosophila*, striatal neurons from LRRK2 KO rats also show slowed endocytosis (Arranz et al., 2015). In contrast, we observe accelerated endocytosis in the LRRK2 KO. Although we used the same striatal cultures and stimulation frequency, we imaged mouse rather than rat neurons, and included a small number of cortical neurons that provide excitatory drive and may make the networks more robust (Penrod et al., 2015). We also used VGAT-pHluorin rather than synaptophysin- and synaptobrevin-pHluorin (Shin et al., 2008; Arranz et al., 2015), although these reporters generally behave in very similar ways. In addition, we have varied a number of conditions, demonstrating that the observed increase in endocytosis occurs across multiple stimulation frequencies, but not in hippocampal neurons which express less LRRK2. It nonetheless remains possible that the role for both phosphorylation (by LRRK2) and dephosphorylation of endophilin proposed by Matta et al. (2012) could result in opposing effects of the KO under different circumstances.

Although the effect of LRRK2 on endocytosis in striatal neurons was only significant with 20 Hz stimulation, stimulation at 10 Hz also shows a trend to faster endocytosis. To investigate a frequency dependence of the effect, we also stimulated with 200 action potentials at 5–40 Hz, and observed faster endocytosis in the KO at all frequencies. Further, stimulation of hippocampal neurons at 20 Hz showed no effect of the KO. We conclude that neuronal identity rather than stimulation frequency is critical to detect an effect of the KO on endocytosis. The work thus indicates a role for endogenous LRRK2 in the slowing of endocytosis. The toxicity asssociated with LRRK2 over-expression makes it difficult to draw conclusions about direct effects, but the phenotype of the KO predicts that a gain of LRRK2 function may also inhibit endocytosis. Consistent with this, loss-of-function mutations in the endocytic proteins synaptojanin and auxilin cause inherited forms of parkinsonism (Edvardson et al., 2012; Quadri et al., 2013). Thus, the inhibition of endocytosis by LRRK2 is consistent with other genetic mechanisms for this form of degeneration.

Although many neuronal populations including midbrain dopamine and hippocampal neurons express LRRK2, high levels of expression in the basal ganglia suggest a particularly important role for striatal LRRK2 in PD (Melrose et al., 2006; Higashi et al., 2007; Westerlund et al., 2008; Giesert et al., 2013). Direct pathway neurons in the striatum project to substantia nigra where they form synapses onto multiple neuronal populations including dopamine neurons (Watabe-Uchida et al., 2012; Calabresi et al., 2014), suggesting that presynaptic LRRK2 might influence striatal input to the midbrain. Alternatively, a disturbance in striatal LRRK2 might exert retrograde effects on presynaptic dopamine terminals in the striatum. Indeed, altered expression of LRRK2 in muscle acts in a retrograde fashion to influence transmitter release at the *Drosophila* neuromuscular junction (Penney et al., 2016), and mutations in LRRK2 lead to increased input from the cortex to striatum (Matikainen-Ankney et al., 2016). Thus, striatal LRRK2 may affect dopaminergic input through a similar, retrograde mechanism.

In conclusion, endogenous LRRK2 normally acts to slow endocytosis in GABAergic striatal neurons but not glutamatergic hippocampal neurons, consistent with the high levels of LRRK2 normally expressed in the striatum and with a non-cell-autonomous role for LRRK2 mutations in the degeneration of dopamine neurons.

## Author Contributions

JWM designed and conducted the imaging experiments, analyzed the data and wrote the manuscript. JY designed and conducted the slice electrophysiology experiments and analyzed the data. RHE oversaw the design of experiments and data analysis, and wrote the manuscript.

## Funding

This work was supported by the following awards: Bumpus Foundation Postdoctoral Fellowship, Wheeler Center Fellowship and the McCamish Young Investigator Award from the National Parkinson Foundation to JWM; Grant no. R01 NS062715 from National Institutes of Health (NIH), a grant from the UCSF Weill Institute for Neurosciences and the John and Helen Cahill Family Endowment for Research on PD to RHE.

## Conflict of Interest Statement

The authors declare that the research was conducted in the absence of any commercial or financial relationships that could be construed as a potential conflict of interest.
